# Contrasting views of animal healthcare providers on worm control practices for sheep and goats in an arid environment

**DOI:** 10.1051/parasite/2012191053

**Published:** 2012-02-15

**Authors:** H.A. Saddiqi, A. Jabbar, W. Babar, M. Sarwar, Z. Iqbal, J. Cabaret

**Affiliations:** 1 Department of Parasitology, University of Agriculture Faisalabad Pakistan; 2 Department of Zoology, Government College University Faisalabad Pakistan; 3 College of Veterinary and Animal Sciences, The Islamia University of Bahawalpur Pakistan; 4 Institute of Animal Nutrition and Feed Technology, University of Agriculture Faisalabad Pakistan; 5 Institut National de la Recherche Agronomique (INRA), IASP, 213 37380 Nouzilly France

**Keywords:** worm control, small ruminant, questionnaire, animal health advisor, traitement antihelminthique, petit ruminant, questionnaire, conseiller, santé animale

## Abstract

A questionnaire survey was conducted to determine the worm control practices and anthelmintic usage of 150 key respondents involved in sheep and goat production in the arid Thal area of Pakistan. The information was collected by visiting farms, and interviewing the key respondents which included veterinary officers (n = 15), veterinary assistants (n = 51), traditional practitioners (n = 24), and small and large scale sheep/goat farm herders and owners (n = 60). Among all interviewed animal healthcare providers, the veterinary officers had the highest level of awareness of parasitic infection and advocated the use of modern available anthelmintics according to the predefined schedule. The farmers on the other hand, had the lowest level of knowledge about parasitic infections. They used modern anthelmintics at low frequencies (every six months) following an unusual practice of diluting the medicine. Veterinary assistants had a medium level of awareness about the parasitic infections using anthelmintic treatments when they deemed necessary rather than following a predefined treatment schedule. Traditional practitioners were also aware of parasitic infections and used traditional anthelmintics or a combination of the traditional and modern anthelmintics. The animal health providers had a different awareness and knowledge of parasitic infections which resulted in contrasting proposals for its control. The farmers used worm control measures in accordance with their own views and those of animal healthcare advisors, combining modern and traditional treatments. This study provides the first insight into the differing views of those animal healthcare providers who form the basis for effective parasitic control within the sheep and goat industry of an arid region.

## Abstract (fr)

Une enquête par questionnaire a été conduite pour étudier les manières de gérer le parasitisme interne et l’utilisation des antihelminthiques chez 150 acteurs de l’élevage des petits ruminants dans la région aride de Thal au Pakistan. L’information était obtenue au cours de visite des fermes avec entretien impliquant les acteurs de la santé que sont les vétérinaires (n = 15), les assistants vétérinaires (n = 51), les praticiens traditionnels (n = 24) et les propriétaires ou bergers des fermes de grande et petite taille (n = 60). Parmi les acteurs de la santé, les vétérinaires ont le plus haut niveau de sensibilisation sur les infections parasitaires et ils proposent des traitements antihelminthiques modernes selon un schéma de contrôle préétabli, alors que les éleveurs présentent une très faible conscience de l’importance du parasitisme. Ces derniers utilisent des antihelminthiques modernes à faible fréquence (tous les six mois) et avec une pratique inhabituelle, celle de diluer ces produits. Les assistants vétérinaires ont une sensibilisation intermédiaire aux problèmes de parasitisme interne et utilisent les antihelminthiques sans plan prédéfini, à la demande. Les praticiens traditionnels ont aussi conscience du risque parasitaire et utilisent aussi bien les traitements modernes que traditionnels. Ainsi, les conseillers pour la santé animale ont des visions différentes du problème parasitaire et proposent donc des solutions assez contrastées. Les éleveurs ont des pratiques de contrôle du parasitisme interne qui résultent de la combinaison de leurs propres opinions et de celles de leurs conseillers. Ce travail met pour la première fois en lumière les visions de quatre acteurs différents de la santé vétérinaire dans un même contexte géographique, qui peuvent ainsi former les bases pour un contrôle plus efficace du parasitisme interne des ovins et caprins.

## Introduction

Helminths are recognized as a major threat to sheep and goat farming in temperate (Cabaret *et al.*, 1986; Cabaret *et al.*, 2009), tropical and arid countries (Mandonnet *et al.*, 2003). In Pakistan, the reported prevalence of gastrointestinal nematodes in (farm) animals is very high (25.1 to 92 %) (Durrani *et al.*, 1981; Iqbal *et al.*, 1993; Qayyum, 1996; Lateef *et al.*, 2005; Raza *et al.*, 2007). The adverse effects of nematode infections include: loss of weight, anorexia, anaemia, retarded growth, delayed sexual maturity, decrease in milk and meat production and an increased susceptibility to secondary illness resulting in considerable production losses (Holmes, 1986; Sykes, 1994; Iqbal *et al.*, 1993). These adverse effects are often the only indication about the presence of infection as the laboratory diagnosis based on nematode egg counts is rarely used, even in developed countries (Cabaret *et al.*, 2009).

The control of gastrointestinal worms in livestock has, for some decades, been largely based on the use of anthelmintics. It has been a profitable choice as it results in a significant increases in milk yield (9 %) and growth rates in sheep (Juste jordan and García perez, 1991), however, due to the development of anthelmintic resistance (Jabbar *et al.*, 2006), the anthelmintics must be used cautiously. The presence of poor-quality drugs has been documented in veterinary (Monteiro *et al.*, 1998; Shakoor *et al.*, 1997; Saddiqi *et al.*, 2006) as well as human medicine (Cabaret, 2010). In Pakistan, as in many resource-poor countries, dilution of drugs, faulty administration practices and miscalculations and/or unawareness about the correct dosage contribute to the low efficacy of anthelmintics (Saeed *et al*., 2007, 2010). Following treatment with modern anthelmintics, a reduced productivity may occur due to either misdiagnosis (the worms are not the cause of the diseases), poor-quality drugs, faulty uses, or resistance of nematodes to the anthelmintics (Jabbar *et al*., 2006, 2008). For those healthcare professionals who lack understanding on the correct identification and recommended treatment of nematode infection, treatment decisions will be largely founded on their beliefs and trust in their counselors. Socio-cultural factors are known to play a pivotal role in the proper implementation of control programs against various infectious diseases (Whiteford, 1997; Yoder, 1997). For example, a study into the health beliefs of people from Nepal demonstrated a strong lack of understanding into the cause and transmission of worm infections (Williams-Blangero *et al*., 1998). Their frequent inability to confirm the efficacy of drug therapy by observing worms in stools has led to a dissatisfaction with biomedical approaches and may cause them to revert back to traditional medicine. The compliance with recommendations of health services may be poor in many circumstances (Cabaret, 2010). A similar situation exists in gastrointestinal worm infections of sheep and goats in Europe and North Africa where farmers do not have a clear picture about the intensity of infection (Cabaret *et al.*, 1986; Berrag *et al.*, 2009) and they also forgo to follow-through with the treatment recommendations provided by extension workers, especially in sheep and goats production (Cabaret, 2003). This kind of breach between the technical recommendations issued by veterinarians or veterinary assistants and their acceptance by farmers is a reality in developed countries (Cabaret, 2003). It may be even more complex in developing countries where the information may arise from different counselors such as veterinarians, veterinary assistants or traditional practitioners (Tsey, 1997). The farmers (herders, owners or shepherds) can also treat their animals based on their own knowledge and beliefs (Nag *et al.*, 2007; Galav *et al.*, 2010) as well as the information obtained following their interaction with the different counselors. A study in Malawi (Hüttner *et al.*, 2001) showed that the biomedical health programs were not accepted by all the farmers, and they did not apply western medicine instead preferring to use local remedies.

In sheep and goat farming, different healthcare providers may have varying views to a particular disease but their knowledge could be profitable to build a common understanding that may result in the better control of diseases. The simultaneous presence of veterinarians, veterinary assistants, traditional practitioners, and farmers of flocks in Pakistan, make the country particularly interesting to compare the views of the different professionals. Furthermore, the role of livestock in the rural economy is important since 30-35 million of the rural population is engaged in livestock raising, having household holdings of twothree cattle/buffalo and five-six sheep/goat per family from which they derive 30-40 % of their income. This is the first study to report the views of four different healthcare providers pertaining to the worm control practices and the use of anthelmintics on small-scale private sheep/goat farms.

## Material and Methods

### Study area and animal characteristics

The locale of the present study was the Thal area (Punjab, Pakistan), which has an arid climate with very hot summers and mild winters. The mean daily temperature ranges from 7 to 41 °C (average 23 °C) and the monthly rainfall varies from 32 (north) to 46 mm (south) except in the winter, which is predominantly dry. The Köppen classification of climates (Viers & Vigneau, 1990) indicates that the Thal arid area is steppic (rainfall less than 74 mm)-desertic (rainfall less than 37 mm). Thal region sprawls over six districts viz., Jang, Khushab, Mianwali, Muzzafar Garh, Bhakkar and Laiyah. For the present investigation, two districts, i.e. Jang and Laiyah, were selected for study. Most of the farmers lived within these Thal arid areas due to the presence of a Government Livestock Experiment Station, Rakh Khairewala, located at the junction of the two districts, which may have been a source of comparison for farmers.

### Farms and data collection

A total of 150 respondents were arbitrarily selected and information was collected by visiting the farms and interviewing the key respondents which included veterinary officers (n = 15), veterinary assistants (n = 51), traditional practitioners (n = 24), and small and large scale sheep/goat farmers (n = 60). The flock sizes ranged from 30 to 120 sheep and goats of mixed breeds. Information on worm control practices and anthelmintic usage was collected through key informant interviews and a questionnaire survey. The first section dealt with anthelmintic usage and aimed at establishing whether animals were drenched and if so, the frequency and occasions for drenching. The main information sought was the level of awareness about parasitic diseases, worm control methods practised, types of anthelmintic used, source of anthelmintics, frequency of treatment and interruption of pre-planned treatments. In addition, questions were asked about alternation/change of anthelmintic classes, source of information on anthelmintic usage, ethnoveterinary medicine and knowledge on sources of worm infection in small ruminants. Information concerning the management of the farms was also noted.

The participating respondents were asked to rank their preference for various anthelmintic preparations and to indicate their criteria for the selection of anthelmintics. They were also asked about the anthelmintics used previously from a list of anthelmintics available in the country. For the question on anthelmintics used, the most commonly used anthelmintics were grouped into three classes. Class I consisted of the benzimidazoles (BZs): oxfendazole, fenbendazole (FBZ), thiabendazole (TBZ), and albendazole (ALB). Levamisole (LEV) and ivermectin (IVM) were grouped as Class II and III, respectively.

### Statistical analyses

Data on the farm characteristics were nominal (Table I). Differences in prevalence were analysed with Chisquare or Fisher exact-test depending on the size of samples. Most of the variables describing the farms are inter-related and global analyses were based on cluster analyses. Each cluster analysis was described in a den- drogram based on an unweighted pair group method with arithmetic average (UPGMA). The dendrograms were constructed with Jaccard coefficient, which is adapted to nominal data (Multivariate statistical package- MVSP 3.1, 2001). Correspondence analysis was done on the responses of farmers/owners, since they constitute a large group in the survey and have the final decision on whether to treat their flock or not. Similarly, correspondence analysis was also performed on the veterinary assistants as they constitute the major source of information for farmers.

**Table I.** Education, awareness and management of internal parasitic diseases of small ruminants in relation to the role of healthcare providers.

	Level/Type/Practice	Veterinary officer (Vetof) N = 15	Veterinary assistant (Veta) N = 51	Traditional practitioner (Tradprac) N = 24	Owner (Farmer) N = 60
Education	ILLITERATE	0.00 ^a^*	0.00 ^a^	0.25 ^b^	0.72 ^c^
	EDUPRIM - Primary education	0.00 ^a^	0.00 ^a^	0.58 ^c^	0.28 ^b^
	EDUSEC — Secondary education	0.00 ^a^	0.43 ^a^	0.17 ^b^	0.00 ^a^
	EDUINT — Intermediate education	0.00 ^a^	0.57 ^b^	0.00 ^a^	0.00 ^a^
	EDUGRAD — Graduate	0.53 ^b^	0.00 ^a^	0.00 ^a^	0.00 ^a^
	EDUPOSTG — Post-graduate	0.47 ^b^	0.00 ^a^	0.00 ^a^	0.00 ^a^

Awareness on internal parasites	LOWAWP — Low awareness	0.00 ^a^	0.12 ^a^	0.00 ^a^	0.25 ^b^
	MediumawP — Medium awareness	0.00 ^a^	0.71 ^c^	0.37 ^b^	0.50 ^b^
	HIGHAWP — High awareness	1.00 ^c^	0.18 ^a^	0.63 ^b^	0.25 ^a^

Management of internal parasites	MODHELM — Use of modern anthelmintics	1.00 ^b^	0.82 ^b^	0.25 ^a^	0.85 ^b^
	TRADHELM — Use of traditional anthelmintics	0.00 ^a^	0.00 ^a^	0.25 ^b^	0.00 ^a^
	Bothtradmod — Use of both	0.00 ^a^	0.18 ^b^	0.50 ^c^	0.15 ^b^
	PREFBZ — Preference for benzimidazole	0.00 ^a^	0.24 ^b^	0.13 ^a^	0.10 ^a^
	PREFLEV — Preference for levamisole	0.40 ^a^	0.41 ^a^	0.50 ^a^	0.80 ^b^
	BOTHANTELM — Use both	0.60 ^c^	0.35 ^b^	0.38 ^b^	0.10 ^a^
	ROTAHELM — Rotation between anthelmintics	0.20 ^c^	0.18 ^c^	0.00 ^a^	0.10 ^b^
	3MOANTH — Anthelmintics every 3 months	0.40 ^b^	0.12 ^a^	0.25 ^a^	0.20 ^a^
	6MOANTH — Anthelmintic every 6 months	0.60 ^a^	0.47 ^a^	0.50 ^a^	0.50 ^a^
	NEEDHELM — Anthelmintics when needed	0.00 ^a^	0.41 ^b^	0.25 ^b^	0.30 ^b^
	DILDRUG — Dilution of the drug	0.60 ^a^	0.35 ^a^	0.63 ^a^	0.75 ^b^

* The different superscript in each row indicates significantly different values at p < 0.05 using Chi-square or Fisher exact test. N indicates the number of each type of respondent.

## Results

### Housing and grazing management

During the summer season, most of the farmers kept their animals in an open environment at night with artificial fences around them. In winter, they were kept in mud houses. Lambs and kids were kept in separate pens to prevent suckling at night. Some farmers (23 %) offered food concentrate to their animals, but the majority of them depended on the available green fodder to graze in open arid areas. Mostly, the animals owned by small-scale farmers grazed together with other animal species (buffalo, cow and goats) present on the farm, whereas those of large-scale farmers grazed separate from other animal species. Lambs and kids were usually kept in fenced yards. They were let out to graze only if the farmer considered them to be capable of doing so without any help. Animals grazed on the banks of rivers/canals and in gram/ wheat harvested fields. Cleanliness on most of the farms (72 %) was very poor; however due to the soiling of the premises, attempts were made to clean the animal yards daily in winter and weekly in summer. The collected dung was used as animal fertilizer in their fields.

### Level of education and awareness about parasitic diseases

The majority of owners (72 %) and some traditional practitioners (25 %) were illiterate while veterinary assistants and officers had a secondary school certificate or a higher secondary school certificate and graduate/post-graduate diploma, respectively (Table I). About half of the respondents had a medium (50 %) level of awareness about parasitic disease, followed by low (36 %) and high (14 %). The highest level of awareness was not completely related to education, and was found among veterinary officers and traditional practitioners (Table I). The majority of the respondents (%) had a limited knowledge about the life cycle and epidemiology of the internal parasites.

### Use of traditional or modern anthelmintics

Most of the respondents were found to use modern anthelmintics (76 %) followed by a mix of both traditional and modern (20 %) and only traditional anthelmintics (4 %). Traditional practitioners mostly used both modern and traditional anthelmintics (50 %), whereas veterinary officers and assistants and owners preferred to use modern anthelmintics. Traditional practitioners and farmers used indigenous homeopathic preparations, which according to them had good results (visual observation for the removal of worms). Herbal or homeopathic dewormers are available from veterinary pharmacies (“Canizole”, against intestinal worms and flukes of sheep and horses; “Deworming plus” against intestinal worms, flukes and external parasites of sheep and horses; “Granil” a combination of dewormer, minerals, vitamins and active enzymes) although these products are not registered in the country.

It was also found that the most of respondents preferred Nilzan plus^®^ (levamisole) and Systamex^®^ (oxfendazole, a benzimidazole). The preference was for those drugs, which initiated diarrhea, such as levamisole, believing that the diarrhea helped to expel the worms. The majority of respondents did not rotate the dewormers (a recommendation to reduce the development of parasite resistance against the drugs) neither did veterinary officers and veterinary assistants. They changed the dewormer only after one drug showed poor results.

### Preference of modern anthelmintics and use of ethnobotanicals

Among the modern synthetic anthelmintics, class II (LEV) was found to be the drug of choice (58 %) followed by class I (BZ) (14 %) and combination of these two classes (28 %). Most of the respondents believed that traditional anthelmintics had no lasting effect or consistent activity. In addition, respondents did not know about the proper dose, duration and repetition of these products. The crude powder or crude water extracts either used singly or in combination with different ethnobotanicals (e.g. *Ferula asafoetida* L., *Azadirachta indica* A. Juss., and *Mallotus philippinensis* Muell.) were found to be used against worms.

### Efficacy of different anthelmintics and frequency of anthelmintic used

There was a great controversy among respondents about the efficacy of different anthelmintics. Mostly, all the respondents used BZ and LEV for the treatment and control of parasitic diseases. Some claimed that the BZ group had better results than LEV, whereas IVM was not used for the treatment of gastrointestinal nematodes, rather it was considered as a drug against mange. Most of the respondents were found to use deworming after every six month (50 %) followed by every three months (20 %) or as when indicated (30 %). Veterinary officers were found to practice tactical deworming based on the epidemiology of helminth infections in arid environments, however some of them recommended deworming after three or six months. Veterinary officers did not consider deworming as the only solution of helminth infections (Table I), however, it was largely used by 25 to 41 % of veterinary assistants, traditional practitioners and farmers. The interruption in pre-planned treatment was practised when a particular dewormer either failed to show satisfactory results or when practitioners introduced a new dewormer in a particular area. The practitioners (veterinary officers and veterinary assistants), medical store keepers and vaccinators were the main source of information on anthelmintics for the farmers. Farmers kept the bottles or leaflets of dewormers for reference and purchased them again when the need arose.

### Diagnosis, dose rate calculation and administration practice

Almost all of the respondents diagnosed parasitic problems by using observable behavioural signs and symptoms. The farmers were aware that diarrhea, poor growth, poor body condition and reduced appetite may indicate parasitic problems. The data revealed that most of the farmers as well as traditional practitioners set roughly two doses, one for adult animals while other for young ones. The veterinarians advised the recommended dose set by the company or overdosed to get good results. The majority of respondents (58 %) administered anthelmintics in diluted form. Veterinary officers (60 %) were also found using diluted drugs for the satisfaction of their clients who suspected that administration of the pure (recommended) drug may lead to toxicity.

### Contrasting strategies for animal healthcare providers

**Fig. 1.**
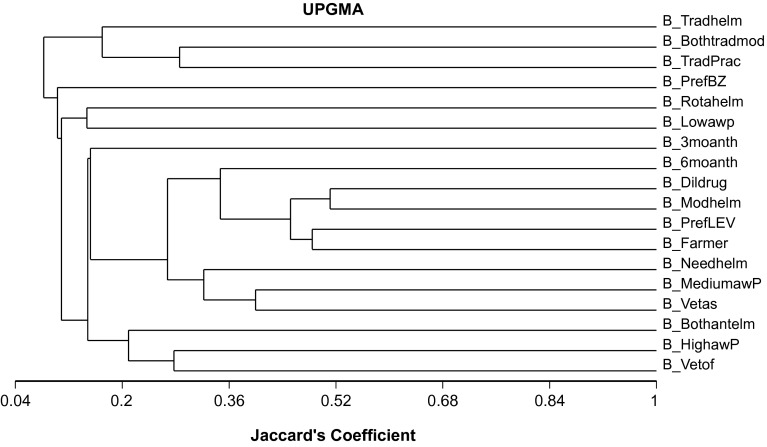
Dendogram (based on cluster analysis using unweighted pair group method with arithmetic average [UPGMA]) showing the management of internal parasites (see Table I for codes) associated with different healthcare providers (veterinary officer: Vetof; veterinary assistant: Vetas; traditional practitioners: Tradprac; and farmers).

**Fig. 2.**
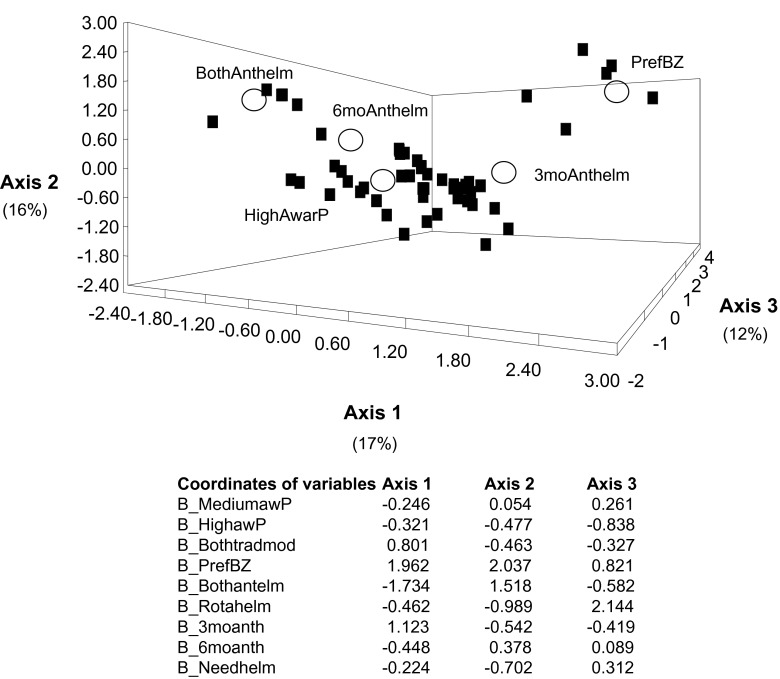
Correspondence analysis case scores for individual responses of farmers (filled squares) in relation to anthelmintic administrations (empty circles). The detail of codes is given in Table I.

**Fig. 3.**
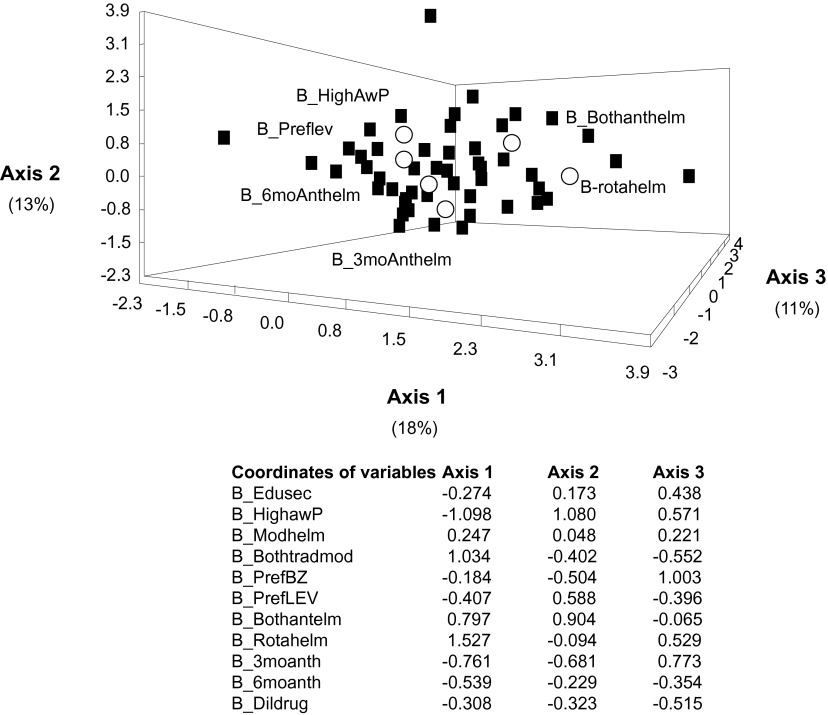
– Correspondence analysis case scores for individual responses of veterinary assistants (filled squares) in relation to anthelmintic prescriptions. The detail of codes is given in Table I.

Figure 1 gives an account of the various anthemlmintic strategies employed by the healthcare providers. Veterinary officers were well-aware of parasitic infection and they advocated the use of both classes (I and II) of available anthelmintics. Veterinary assistants had a medium level of awareness about the parasitic infections and they used the treatment when needed rather than in accordance with a predefined treatment schedule. Traditional practitioners used traditional anthelmintics or a combination of traditional and modern anthelmintics. Farmers preferred anthelmintics, especially LEV, and they used it every six months following their dilution. There was some variability among the farmers (Fig. 2) where the first axis of the correspondence analysis was well represented by the preference of BZ as well as the use of both types of modern anthelmintics and treatment of animals after every three months. The second axis was represented by the preference for BZs or use of both modern anthelmintics and rotation between the two modern anthelmintics. A small group was characterized by the preference for BZs and treatment every three months, which was in contrast to another small group with the use of both anthelmintics and treatment every six months. A similar analysis of veterinary assistants showed a different pattern (Fig. 3) where the first axis showed the high awareness of parasitic infection, rotation of anthelmintics, use of modern and traditional drugs, and the second axis was represented by high awareness of parasitic infection and the use of both modern anthelmintics (Class I and II). No particular group could be detected, but three veterinary assistants were distinct from others, and had the lowest education level and parasite awareness.

## Discussion

This survey has shown that worm control in sheep and goat production in Thal has mostly been based on the use of anthelmintics. Similar results have been reported elsewhere including Kenya (Kinoti *et al.*, 1994; Maingi *et al.*, 1997) and Brazil (Charles & Furlong, 1996). Modern anthelmintic treatments used by the respondents interviewed herein depended on the availability of money or drugs and not the epidemiology of parasites. In most developing countries, treatment choices are based on the availability of money rather than baseline data regarding epidemiology of helminths. In our survey, the maximum deworming was carried out in the month of April followed by May/June, July, September and February. The use of anthelmintics in different months of the year did not indicate any strategic deworming, rather it was based on the appearance of signs of parasitic diseases or on the availability of anthelmintics. The climatic conditions of July, August and September are highly favorable for the propagation of infective larvae of nematode species prevalent in the study area (Lateef *et al.*, 2005). The decision to treat, although constrained by biology and resources, is very much reliant on how the healthcare providers detect the gastrointestinal nematodes and their beliefs on the best way to treat animals against these parasites.

Salmona (1994) described that the decisions in agriculture are based on several types of knowledge i.e., algorithmic (learnt in books/school/journals), mimetic (learnt from a demonstration) and phoric (what one feels). The weights of these types of knowledge are probably very different in the decision of the various healthcare providers. Knowledge is one part of the decision but values or economic resources may play a role as well (Cabaret *et al.*, 2009). We intended to evaluate the intensity of controversy on parasite management and its reasons in the Thal region of Pakistan, where four animal healthcare providers are present, representing a common situation in developing countries. The gastrointestinal nematode infection is a good system to evaluate the construction of infection control, since no empirical evaluation (e.g. gastrointestinal nematode eggs per gram of host faeces) is done in this area and control is based on the animals’ symptoms (diarrhea, anemia) and production traits.

The veterinary officers’ choices for the anthelmintic treatments depend on algorithmic knowledge. They were taught according to the older textbooks that: “*the animals should be treated regularly in order to keep them free from the most harmful worms; so that they will be able to overcome the parasites*” (Soulsby, 1968); it translates with the promotion of frequent treatments of the whole flock. In a recent textbook (Kilani *et al.*, 2010), it was reported that “*currently, the use of anthelmintics either in chemoprophylaxis programmes or as periodic treatments, remains the principal means of control of gastrointestinal strongyloses in ruminants and the timing and intervals between treatments of the flock/herd should be established strategically and the general rules designed to prevent or slow down the occurrence of anthelmintic resistance should be also observed*”. This means that we rely fully on regular anthelmintic use (and thus based on modern anthelmintics only) for the control of gastrointestinal nematodes, but we need to avoid those practices (reviewed by Jabbar *et al.*, 2006), which may favour the development of anthelmintic resistance. The importance of gastrointestinal nematodes on health and performances is recorded in textbooks and may explain the high awareness of veterinary officers about parasites. Veterinarians are usually called upon after the traditional practitioners have been unsuccessful, when the animals’ condition has worsened. Veterinarians’ views are highly different from those of traditional practitioners and a previous study in India showed that veterinarians perceived traditional practitioners as the main (53.75 %) constraint related to disease prevention followed by the farmers (46.25 %) (Venkatasubramanian & Fulzele, 1996).

The veterinary assistants are closely related with the veterinary officers (algorithmic knowledge) and farmers with different knowledge as they are largely illiterate. However, the veterinary assistants show differences in their views compared with veterinary officer; their awareness of parasites is much lower and comparable to that of farmers and they prefer to use BZs and do not practice the alternation of drugs. Unlike veterinary officers but like farmers, veterinary assistants use more traditional anthelmintics and when they use modern anthelmintics, usually combine them with traditional anthelmintics. They accept the strategy of treating animals only in need of treatment in contrast with the practice of veterinary officers. Thus, their knowledge, although it was expected to be very similar to that experienced by veterinary officers (algorithmic), is very different – possibly due to the frequent exchanges with farmers on the subject of parasite control as they spend more of their time in the field than veterinary officers.

The views of the traditional practitioners differed the most. They rely on the traditional anthelmintics or a combination of modern and traditional drugs. Their knowledge is a mixture of the algorithmic (capacity to read, use of modern anthelmintics), the mimetic (learn from someone who knew medicinal plants and other ingredients) and possibly phoric (what they feel). Herbal resources are large and known from traditional practitioners and partly by farmers. Nag *et al.* (2007) reported that about 30 diseases of domestic animals in Rajahstan could be treated by 62 plant species found in the local vicinity. Traditional practitioners have an unexpectedly high level of awareness of internal parasites (63 %) compared to veterinary assistants and farmers (18-25 %).

In the present study, we found that the majority (72 %) of the farmers were illiterate and they found it difficult to gather and maintain information on what they should do, thus explaining why they kept the bottles and leaflets of dewormers for the future selfprescribed medication of their animals. A similar attitude was observed in farmers in Morocco; when farmers were advised to decrease the number of anthelmintic treatments, they agreed to do so, but requested guidance (for ?) even on their own farm(s) (Berrag *et al.*, 2009). Farmers in this study had a preference for levamisole and surprisingly, never solely relied upon traditional anthelmintics. This is in contrast with the previous reports from the same country (Nag *et al.*, 2007; Galav *et al.*, 2010), where farmers have their own bases for treating the animals. In livestock-rearing communities in different parts of the Indian states of Andhra Pradesh and Maharashtra, the communal knowledge on ethnoveterinary products is an integral part of the management practices of farmers, and over 80 % of the farmers continue to use such products as they are cheap and easily available, especially in remote villages (Ghotge *et al.*, 2002). In the present study, the use of local/ethnobotanical remedies for worm control was rare which might be due to the presence of a livestock experiment station in the area where only modern anthelmintics had been used since its establishment.

A comparison of the views of individual animal healthcare providers acting in the field was done on the veterinary assistants and farmers, as they had the largest numbers and allowed a comparison. Several groups could be detected among farmers; whereas only individual particularities were evidenced among veterinary assistants. This could mean that the veterinary assistants were taught the same management of regulating gastrointestinal nematodes (only three of them were unusual, possibly in relation to limited education) whereas the farmers were distributed into several groups, since they rely mostly upon mimetic (advices from veterinary officers and assistants or traditional practitioners) and phoretic knowledge (what they believe to be good for their flock).

The present study provides the first report about the views of different healthcare providers for the gastrointestinal nematode control practices in sheep and goat production. The data reported herein suggest that the scientific knowledge is not being transferred to the end-users. The transfer of knowledge not only involves the transmission of the technical information but it also includes the dissemination of the cultural values for the successful completion of a disease control program. The study revealed that each group of the animal healthcare providers has its own vision of how to control gastrointestinal nematodes of sheep and goat and even within the farmers, several visions could be identified. It would be interesting to understand why farmers were a more variable group as it may result in more effective proposals for anthelmintic treatments.
